# Hypopigmented mycosis fungoides: an important differential diagnosis of hypochromias in childhood

**DOI:** 10.1590/1984-0462/2025/43/2024181

**Published:** 2025-01-17

**Authors:** Maria Fernanda de Almeida Cavalcante Aranha, Maria Amélia Lopes dos Santos, Carla Andréa Avelar Pires, Leônidas Braga Dias, Rafaela Garcia Pereira, Marina Lopes de Freitas Freire, Tamara Tavares da Cruz, Luiza Rennó Rocha de Oliveira

**Affiliations:** a Universidade do Estado do Pará, Belém, PA, Brazil.

**Keywords:** Mycosis fungoides, Diagnosis, differential, Dermatology, Child, Micose fungoide, Diagnóstico diferencial, Dermatologia, Criança

## Abstract

**Objective:**

To highlight the importance of early recognition of hypopigmented mycosis fungoides (HMF) in cases of cutaneous hypochromia in children, with a view to an effective diagnostic and therapeutic approach.

**Case description:**

Two cases of HMF in children are reported. The first case involves an eight-year-old boy with hypochromic macules on the trunk and root of the upper and lower limbs, while the second case is a six-year-old boy with widespread hypochromic patches. Both patients presented with prolonged evolution of hypopigmentation, leading to the suspicion of HMF after excluding other differential diagnoses. Histopathological and immunohistochemical tests were fundamental in confirming the diagnosis of HMF.

**Comments:**

HMF is a less prevalent and less publicized form of mycosis fungoides and is more common in children and people with a high phototype. Its diagnosis is challenging and often requires multiple biopsies for confirmation. Treatment includes phototherapy and immunosuppressive therapy, depending on the patient’s age and extent of lesions. Early recognition of HMF is crucial for proper management and to avoid complications associated with malignant evolution.

## RESUMO

**Objetivo:**

Destacar a importância do reconhecimento precoce da micose fungoide hipocromiante (MFH) em casos de hipocromia cutânea em crianças, visando a uma abordagem diagnóstica e terapêutica eficazes.

**Descrição do caso:**

Dois casos de MFH em crianças são relatados. O primeiro caso envolve um menino de oito anos com máculas hipocrômicas no tronco e na raiz dos membros superiores e inferiores, enquanto o segundo caso é de um menino de seis anos com manchas hipocrômicas disseminadas. Ambos os pacientes apresentaram evolução prolongada da hipopigmentação, levando à suspeita de MFH após exclusão de outros diagnósticos diferenciais. Os exames histopatológicos e imuno-histoquímicos foram fundamentais para confirmar o diagnóstico de MFH.

**Comentários:**

A MFH é uma forma pouco prevalente e pouco divulgada da micose fungoide, sendo mais comum em crianças e em pessoas de fototipo elevado. Seu diagnóstico é desafiador e muitas vezes requer múltiplas biópsias para confirmação. O tratamento inclui terapias fotodinâmicas e imunossupressoras, dependendo da idade do paciente e da extensão das lesões. O reconhecimento precoce da MFH é crucial para um manejo adequado e para evitar complicações associadas à evolução maligna.

## INTRODUCTION

Mycosis fungoides (MF) is the most common primary cutaneous non-Hodgkin’s T-cell lymphoma. The World Health Organization subdivides it into the classic, pagetoid reticulosis, folliculotropic, and granulomatous slack skin forms.^1^ Hypopigmented mycosis fungoides (HMF) is a variant of the classic form^2-5^ that is most prevalent in young people, accounting for 17 to 59% of MF cases in children, and is more common in African-Americans, Asians, and people of mixed African and Caucasian descent.^6-13^

Clinically, HMF can be expressed as hypochromic macules or erythematosquamous plaques,^14^ asymptomatic or slightly pruritic. The lesions are usually multiple,^14-18^ with a preference for areas not exposed to the sun, such as the trunk, thighs, and buttocks. Other possibilities include lesions accompanied by atrophy, telangiectasia, or even after exposure to the sun. In general, it has an indolent course and rare extra-cutaneous dissemination.^10,14,19^

Regarding pathophysiology, it is believed that when skin homeostasis is lost due to skin damage, keratinocytes release pro-inflammatory cytokines that mobilize innate and adaptive immune cells to the skin tissue. The activated immune cells migrate to the lymph nodes, encounter immature T cells, and activate them.^20^ In HMF, there seems to be an effective immune response resulting from a powerful cytotoxic response, which would explain its occurrence in a young population that shows no signs of immunosenescence. Hypochromia results from a reduced number of melanocytes and abnormal melanogenesis due to the cytotoxic action of CD8^+^ T cells against melanocytes.^10,20^

Diagnosing HMF is challenging due to its clinical-pathological and molecular characteristics;^9^ multiple biopsies are sometimes necessary to confirm it.^21^ HMF has distinctive histopathological and molecular features compared to classic MF in general, such as a higher frequency of CD8^+^ lymphocytes in the epidermis,^6^ in addition to atypical and enlarged lymphocytes, with a halo and convoluted nucleus, contrasting with the mild to moderate dermal infiltrate.^22^ The first-line treatment for this variant is phototherapy.^23^

Two cases of HMF in childhood are reported, and the importance of recognizing the disease and including this diagnostic hypothesis in the context of diseases with hypochromia is discussed.

## CASE REPORT

### Case 1

A male patient, eight years old, phototype V, evolved for two years with hypochromic, slightly pruritic macules, with a progressive increase in the number and size of lesions. The diagnoses of pityriasis alba and pityriasis versicolor were made, and he did not respond to treatment with barrier repair moisturizers, calcineurin inhibitors, or topical antifungals.

**Figure 1:**
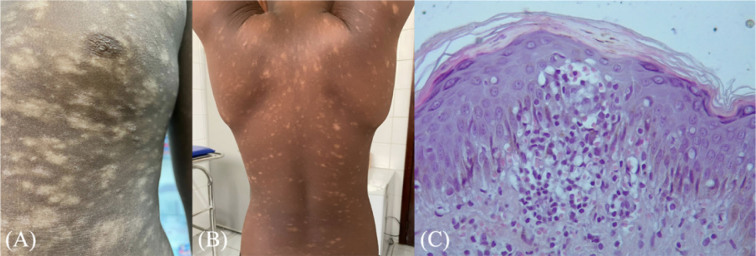
(A and B) Hypochromic macules of varying sizes, numerous, confluent, and coalescent, spread over the trunk and roots of the upper and lower limbs, and cutaneous xerosis. (C) Apparent Darier-Pautrier microabscesses with epidermotropism, dermal infiltrate, and extravasated red blood cells.

Source: personal archive.

Dermatological examination revealed xerosis and irregular hypochromic macules, 0.5 cm to 6.0 cm in size, located on the anterior and posterior trunk, buttocks, and proximal portion of the upper and lower limbs, some confluent and coalescent, interspersed with rare slightly scaly erythematous plaques (Figures 1A and 1B), without altered sensitivity and non-fluorescent under Wood’s lamp.

A biopsy was conducted under suspicion of HMF. The histopathology revealed an epidermis with discrete orthokeratosis, irregular acanthosis, spongiosis, and exocytosis of small, atypical lymphocytes, with the formation of apparent Darier-Pautrier microabscesses compatible with the diagnosis of mycosis fungoides (Figure 1C). This was followed by immunohistochemistry, in which atypical epidermotropic lymphocytes were positive for CD3, CD4, CD8, and KI-67. Other laboratory tests were unchanged.

Treatment with narrowband ultraviolet B (NB-UVB) therapy was indicated, and the patient has been monitored.

### Case 2

A male patient, six years old, phototype IV, evolved for one year with hypochromic macules initially observed on the face, with later craniocaudal progression to other body segments, without associated symptoms.

**Figure 2:**
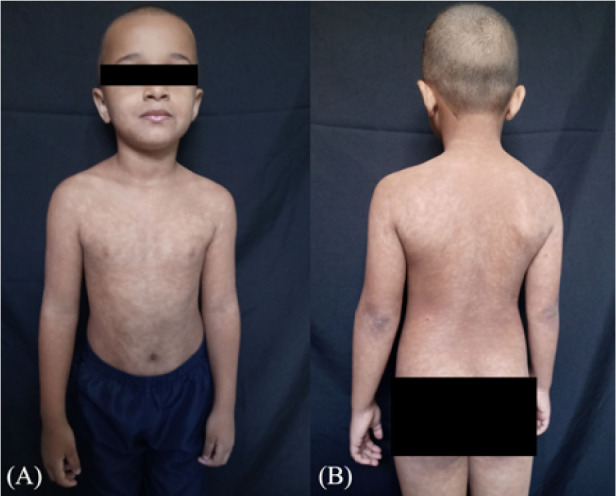
(A and B) Hypochromic macules with unclear boundaries, irregular contours, some confluent, others coalescent, interspersed with healthy skin, distributed throughout the skin, sparing the distal third of the lower limbs.

Source: personal archive.

Dermatological examination revealed irregular hypochromic macules, some confluent, others coalescent, interspersed with healthy skin, distributed throughout the skin (Figure 2), sparing only the distal third of the lower limbs.

Under the hypotheses of pityriasis alba, progressive macular hypomelanosis, and hypopigmented mycosis fungoides, a biopsy was performed. The histopathology was non-specific, but immunohistochemistry showed T lymphoid cellularity with epidermotropism and co-expression of CD4 and CD8, detected by double staining, as well as loss of expression of CD2, CD5, and CD7, favoring the hypothesis of hypopigmented mycosis fungoides. Other laboratory tests were unchanged.

Due to the patient’s unwillingness to undergo phototherapy, home heliotherapy was indicated, with advice to expose him to the sun for ten minutes between 7:30 and 9:00 am. He has been monitored by the service.

## DISCUSSION

Among the dermatological diseases that have hypochromia, although HMF is not prevalent,^2^ it should be considered a diagnostic possibility in children, either because this form can manifest in a young population,^6-9^ or because of the rare chances of a poor prognosis.^9^ Signs of unfavorable evolution of MF include extensive skin involvement, the presence of tumors and erythroderma, and lymph node, visceral, and hematological involvement.^24^

The main differential diagnoses for HMF reported in the medical literature are pityriasis versicolor, pityriasis alba, post-inflammatory hypopigmentation, progressive macular hypomelanosis, and sarcoidosis.^2^ The diagnoses of pityriasis versicolor, pityriasis alba, and progressive macular hypomelanosis were attributed to the cases reported; however, the prolonged evolution, progressive nature, refractoriness to initial therapies, and location of the lesions led to the suspicion of HMF in both cases.

The diagnosis can be challenging to confirm, requiring detailed assessment, follow-up, and complementary tests such as histopathology and immunohistochemistry.^9^ In the face of inconclusive histology, the patient should be followed up as a potential carrier of HMF, as the interval between the appearance of skin lesions and histopathological diagnosis is, on average, two years.^25^ The finding of Darier-Pautrier microabscesses on histopathology and the favorable immunohistochemical pattern for MF in the cases described determined that the hypopigmentation presented by the children corresponds to the hypopigmented form of MF.

Phototherapy is recommended as the first line for HMF. The modality using NB-UVB is administered to children younger than ten, and the association of psoralen plus ultraviolet A (PUVA) therapy is preferred for patients over the age of ten.^23^ NB-UVB was indicated for the first patient and home heliotherapy was recommended for the second due to his unavailability for this treatment.

It should be noted that the hypopigmented form of MF is a clinical presentation of the disease that is not very prevalent or well publicized. Still, its recognition is important due to the possibility of poor outcomes. It is, therefore, important to include this diagnostic hypothesis in cases of cutaneous hypochromia, especially in children, to obtain an appropriate diagnostic and therapeutic approach, ensuring better disease improvement.

## Data Availability

The database that originated the article is available with the corresponding author. CAAE: 80821024.2.0000.5174
